# Long-term trends in the labour supply and productivity of pharmacists in Canada

**DOI:** 10.1177/17151635251330871

**Published:** 2025-06-11

**Authors:** Paul Grootendorst, Boris Kralj, Arthur Sweetman

**Affiliations:** Leslie Dan Faculty of Pharmacy, University of Toronto, Toronto ON; Department of Economics, McMaster University, Hamilton ON; Department of Economics, McMaster University, Hamilton ON; Department of Economics, McMaster University, Hamilton ON

## Abstract

**Background:**

The pharmacist labour supply affects patient access to pharmacotherapy, immunization, and other primary health care services. There is little published evidence on the pharmacist labour supply in Canada, yet these data are needed for pharmacist workforce planning. We evaluated long-term trends in the number of pharmacists in Canada, their average hours worked, and how demographic and other factors affect hours worked. We also examined trends in community pharmacist productivity.

**Methods:**

Data on the number of licensed pharmacists were obtained from pharmacist regulatory agencies. Statistics Canada’s Labour Force Survey identified pharmacists’ hours worked per week to be between 1987 and 2023. Regression models were used to estimate the impact of pharmacist demographic characteristics, worksite type, region of residence, and secular trends on hours worked. IQVIA data on community pharmacist prescription dispensing were used to examine productivity.

**Results:**

The number of pharmacists relative to population doubled over the past 4 decades. This growth was partly offset by reductions in average hours worked per week. This appears to be driven by increases in the female share of the pharmacist workforce and the declining number of hours that male pharmacists work. On net, however, the total number of weekly hours worked—the average weekly hours worked per pharmacist times the number of pharmacists—has increased in both absolute and per capita terms. This expansion in the pharmacist labour supply was reinforced by an increase in pharmacist productivity, at least in the community pharmacy sector.

**Interpretation:**

The pharmacist labour supply in Canada has expanded markedly over the past 4 decades; this has occurred despite a decline in the average weekly hours worked by male pharmacists. It is unclear, however, whether this increase is commensurate with the increased responsibilities and workloads being placed on pharmacists. Province-level data on the forecasted demand for pharmacist services and the pharmacist personnel needed to provide these services are required for pharmacist workforce planning. *Can Pharm J (Ott)* 2025;158:236–246.

**Knowledge into Practice**

- Our study is the first to provide empirical evidence on long-term trends in the number of pharmacists in Canada, their average hours worked, and community pharmacist productivity.
- The pharmacist labour supply in Canada—the total number of hours pharmacists work relative to population—has expanded markedly over the past 4 decades; this has occurred despite a decline in the average weekly hours worked by male pharmacists and larger numbers of female pharmacists who tend to work fewer hours than male pharmacists do.
- Community pharmacist productivity has also increased, possibly due to the increased use of pharmacy technicians.
- Our analysis suggests that the pharmacist labour supply has increased; it is unclear, however, whether this increase is commensurate with the increased responsibilities and workloads being placed on pharmacists.

## Introduction

Pharmacists are playing an increasingly important role in Canada’s health care system. The scope of prescription medications to treat and manage health problems has expanded markedly over the past several decades. So, too, has the need for pharmacists to manage increasingly complex prescription medication regimens. Pharmacists have additionally been granted authority to provide an increasing number of primary health care services such as vaccinations, smoking cessation counselling, and the right to diagnose and prescribe drugs for common ailments.^1^

To ensure an adequate pharmacist workforce in the future, governments require data on various facets of the pharmacist labour supply. These estimates are used to determine, among other things, pharmacy school enrollments and pharmacist immigration targets. Somewhat surprisingly, however, the literature contains very limited evidence on the pharmacist labour supply in Canada.^2^ This article, therefore, sheds light on this issue. We present data on trends in the size of the pharmacist workforce, its demographic composition, and the average number of hours worked. A trend analysis can be used to forecast pharmacist capacity, at least in the short term.

To begin, we present trends in the number of licensed pharmacists per 10,000 population over the period 1982 to 2023. Although the per capita number of health care providers is a useful and commonly reported statistic, it fails to measure all pertinent aspects of provider labour supply. First, it does not capture the weekly number of hours that providers work, which can change over time. For instance, the female share of the pharmacy workforce is steadily increasing.^3^ The Royal Commission on Health Services, also known as the Hall Commission, reported that 11% of the pharmacist workforce was female in 1962.^4^ The female share of the pharmacist workforce increased to 60% by 2022.^3^ Females tend to work fewer hours than males do,^5^ typically because of child-rearing responsibilities. This appears to be true in the medical^6^ and pharmacy^7^ professions as well. Indeed, Goldin and Katz^8^ reported that females have been drawn to the pharmacy profession precisely because of the flexibility in the number of hours worked. Given this, one might expect that the growing ranks of female pharmacists would result in a reduction in the average number of hours worked per pharmacist. In addition to a growing female share of the pharmacy workforce, there is a growing number of foreign-trained pharmacists, known as international pharmacy graduates (IPGs), practicing in Canada.^3^ It is unclear if IPGs work the same number of hours as pharmacists who trained in Canada do.

**Mise En Pratique Des Connaissances**

- Notre étude est la première à fournir des données empiriques sur les tendances à long terme du nombre de pharmaciens au Canada, de leur nombre moyen d’heures travaillées et de la productivité des pharmaciens communautaires.
- L’offre de main-d’œuvre en pharmacie au Canada – le nombre total d’heures travaillées par les pharmaciens par rapport à la population – a considérablement augmenté au cours des 4 dernières décennies, et ce, malgré une baisse de la moyenne hebdomadaire des heures travaillées par les pharmaciens hommes et un plus grand nombre de pharmaciennes qui ont tendance à travailler moins d’heures que les pharmaciens.
- La productivité des pharmaciens communautaires a également augmenté, peut-être en raison du recours accru de techniciens en pharmacie.
- Notre analyse suggère que l’offre de main-d’œuvre en pharmacie a augmenté; il n’est toutefois pas clair si cette augmentation est proportionnelle aux responsabilités et à la charge de travail accrues qui leur sont confiées.

To investigate, we used data from the Statistics Canada Labour Force Survey conducted over the period 1987 to 2023 to present trends in the weekly hours worked by pharmacists and how this varies by sex and by immigrant status (born in Canada vs immigrant; we use the pharmacist’s immigrant status as a proxy for their location of training: international vs domestic). We also estimated linear regression models to understand how average weekly hours worked vary with pharmacist sex, age, province or region of Canada, time period, and workplace (public vs private sector).

The per capita number of health care providers statistic also fails to account for the productivity of health care providers. Pharmacist productivity has likely increased from the adoption of labour-saving technology, such as pill-counting machines, and the growing use of licensed pharmacy technicians, who are allowed to perform the more mechanical aspects of prescription filling. The National Association of Pharmacy Regulatory Authorities (NAPRA) reports that as of January 2024, there were 2376 pharmacy technicians vs 12,856 pharmacists working in community pharmacies in Ontario.^9^ Pharmacy technician use was even more intensive in Ontario hospitals (3443 technicians vs 3007 pharmacists).^9^

We therefore also present data on the trends in the volume of output performed per hour of work by community pharmacists. We focus on community pharmacists, as pharmacists working in this setting account for about 73% of the pharmacy workforce; the remaining 27% work in hospitals and non-clinical settings.^9^ Our measure of productivity is the number of prescriptions dispensed per community pharmacist. Even though pharmacists provide a variety of primary care services in addition to filling prescriptions, the prescriptions per hour worked statistic is an important measure of pharmacist productivity for 2 reasons. First, community pharmacies typically derive most of their revenues from filling prescriptions,^10^ so prescription dispensing and counselling remain core services. Second, there is less scope to deploy labour-saving technologies and the use of allied pharmacy personnel to increase pharmacist productivity in the provision of clinical services, such as medication reviews and common ailment consultations. Thus, one would not expect to see much change in the volume of quality-standardized clinical services a pharmacist provides per hour.

## Methods

### Data sources

National data on the number of licensed pharmacists for the period 2001 to 2023 were obtained from NAPRA. NAPRA, in turn, obtained these data from provincial and territorial pharmacist regulatory authorities, each of which maintains a registry of licensed pharmacists. NAPRA reports pharmacist counts as of January 1. These data were assigned to the previous year; for instance, counts reported on January 1, 2024 were interpreted as counts for 2023. Data on the number of licensed pharmacists for the period 1988 to 2000 were obtained from 2 reports published by the Canadian Institute for Health Information (CIHI), 1 providing data from 1991 to 2000^11^ and the other providing data from 1988 to 1990.^12^ Data for the period 1982 to 1987 were obtained from a report by Health and Welfare Canada.^13^ Note that not all licensed pharmacists provide patient care. According to CIHI, in 2022, about 5% of pharmacists worked in non-clinical settings such as in regulatory agencies, the pharmaceutical industry, and educational institutions.^3^

Our analysis used data on the hours worked per week reported by respondents to Statistics Canada’s Labour Force Survey (LFS), a mandatory monthly survey that sampled about 54,000 households (110,000 people) in 2020.^14^ Respondents were interviewed in 6 successive months, typically mid-month; we employed the first interview for each respondent. We focused on the surveys conducted over the period 1987 to 2023. Appendix 1, in the Supplementary Materials available online, provides details on the LFS and the subset of the LFS we used for our analysis.

We accessed and analyzed the confidential LFS master file through the Statistics Canada Research Data Centre at McMaster University. Statistics Canada maintains the secured Research Data Centres (RDCs) in select universities and government offices across Canada.^15^ Individuals wishing to access data at an RDC must obtain a security clearance, complete mandatory training, and swear or affirm the Oath of Office and Secrecy to Statistics Canada.

To measure the prescriptions dispensed per community pharmacist, we obtained IQVIA data on the number of prescriptions dispensed annually in community pharmacies over the period 1995 to 2023. IQVIA (formerly IMS Health) is a leading provider of pharmaceutical market sales data. We used data from IQVIA’s CompuScript survey; this survey collects dispensing data from a sample of roughly 30% of community pharmacies from across the 10 provinces.^16^ NAPRA provided data on the number of pharmacists working in community pharmacies and the number of community pharmacies in the 10 provinces for the period 2001 to 2023. Territorial counts were excluded since the prescription volume data we used to measure productivity excluded the territories. During the period 2006 to 2021, community pharmacist counts for Alberta were unavailable; data on total licensed pharmacists (across all workplace settings) were reported. We estimated community pharmacist counts for Alberta for these years by estimating, for each year, the average community pharmacist share of the total licensed pharmacist workforce in the other 9 provinces and then multiplying this share by the total number of pharmacists in Alberta. Data on community pharmacist and pharmacy counts for the years 1999 to 2000 were obtained from the Community Pharmacy Trends Report produced by Rogers Media.^17^ Annual national population counts were obtained from Statistics Canada.^18^

### Data analysis

We prepared a graph of the pharmacist per population ratios over the period 1982 to 2023 and community pharmacist per population ratios over the period 1999 to 2023. We graphed the average hours worked per week by pharmacists of normal working age (23–64 years) for each year over the period 1987 to 2023. These averages employed the LFS sampling weights provided by Statistics Canada. A limitation with the use of the LFS data is that pharmacists constitute only about 0.1% of the Canadian population. The LFS excludes some groups from its sampling frame—those who are institutionalized, those younger than 15 years, and some other groups—but even with this adjustment, the number of pharmacists who appear in the LFS is small, and this reduces the precision of the average. To smooth these fluctuations, we fit a median spline curve to the annual estimates to reveal local trends. We also produced graphs of trends of average pharmacist weekly work hours by sex (male vs female) and by immigrant status (born in Canada, born outside Canada). We expect that LFS observations on pharmacists who immigrated to Canada would in most cases be IPGs, but there are some exceptions. These include individuals born in Canada who obtained their pharmacy education outside of Canada and individuals who immigrated to Canada at a young age and subsequently obtained their pharmacy education within Canada. Finally, we produced graphs of the average number of prescriptions dispensed per community pharmacist and per community pharmacy over the period 1999 to 2023.

We estimated the linear regression models of the average weekly hours of work using the LFS data on individuals aged 23 to 80 years. Model parameters were estimated using ordinary least squares and LFS weights. Heteroskedasticity-consistent standard errors were used to estimate 95% confidence intervals for model parameters. All analyses were conducted using Stata version 17.0.^19^

The models included covariates to capture pharmacist demographic characteristics, geographic region of residence, survey period, and workplace type. The demographic covariates consisted of indicators of age group, male sex, and the presence of children younger than 5 years at home. The age groups were 31 to 40, 41 to 50, 51 to 60, 61 to 70, and 70+ years; 30 years and younger was the reference group. We used indicators for the following province or region of residence: British Columbia, Alberta, the Prairies (Saskatchewan, Manitoba), Ontario, and Quebec; the Atlantic region (New Brunswick, Nova Scotia, Prince Edward Island, Newfoundland) constituted the reference category.

We used indicators of the following 5-year survey periods: 1994 to 1998, 1999 to 2003, 2004 to 2008, 2009 to 2013, 2014 to 2018, and 2019 to 2023; 1987 to 1993 was the reference period. The LFS data distinguish respondents who work in publicly owned facilities, those who are employees of privately owned businesses, and those who are self-employed. We constructed indicators for each of the latter 2 categories; employees of publicly owned facilities formed the reference group. Pharmacists who are public facility employees typically work in hospitals (hospitals, with very limited exceptions, are either publicly owned or run by private not-for-profit corporations^20^). Pharmacist employees of privately owned businesses typically work in a community pharmacy; a small fraction work in the pharmaceutical industry or in other sectors. Self-employed pharmacists typically own a pharmacy; in some cases, they own a professional corporation that is contracted to operate a corporate chain pharmacy. The models were re-estimated separately for males and females to assess whether the effects of other covariates varied by sex.

### Ethics approval

Our analysis did not require ethics review board approval because the LFS data were analyzed within an RDC. The other data we rely on are publicly available.

## Results

**Figure 1:**
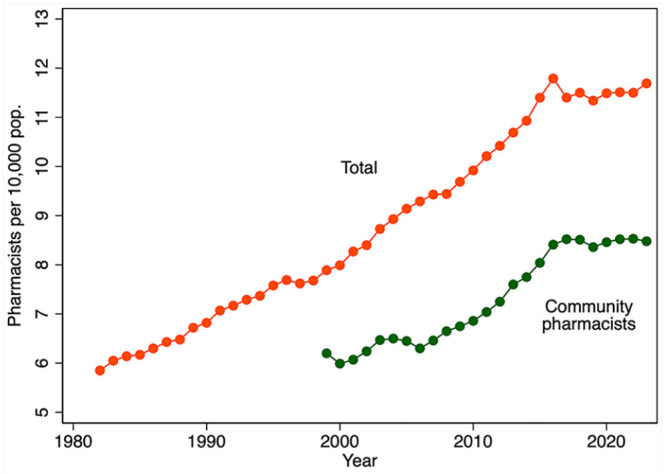
Licensed pharmacists per 10,000 population, by year, 1982–2023 (total) and 1999–2023 (community pharmacists) Pop., population. Data source: Total pharmacists, 2001–2023: National Association of Pharmacy Regulatory Authorities (NAPRA).^9^ Community pharmacists, 2001-2023: NAPRA.^9^ Pre-2001: Canadian Institute for Health Information and Health and Welfare Canada.^11-13^ 1999-2000: Community Pharmacy Trends Report by Healthcare and Financial Publishing.^17^ Population: Statistics Canada Table: 17-10-0005-01.^18^

**Figure 2:**
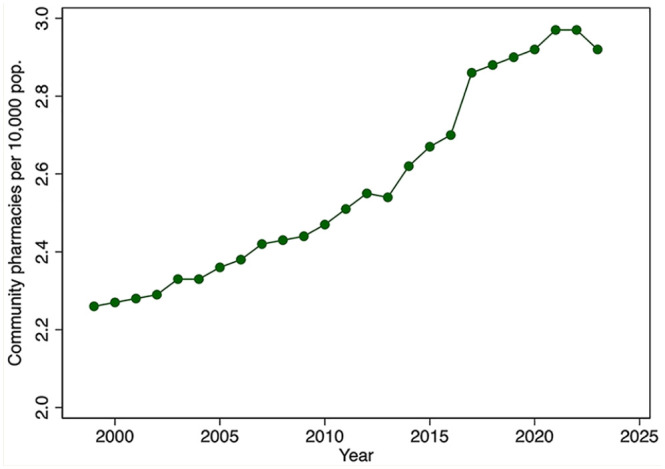
Community pharmacies per 10,000 population, by year, 1999–2023 Pop., population. Data source: Community pharmacies, 2001–2023: NAPRA.^9^ 1999-2000: Community Pharmacy Trends Report by Healthcare and Financial Publishing.^17^ Population: Statistics Canada Table: 17-10-0005-01.^18^

There has been a marked increase in the per capita number of pharmacists and the number of pharmacists working in community pharmacies over the past several decades (Figure 1). The total number of pharmacists per population doubled from 1982 to 2015 and stabilized thereafter. The number of community pharmacists per capita increased by about 40% during the period 1999 to 2023. Figure 2 shows a similar expansion of the number of community pharmacies per 10,000 population, by year, 1999 to 2023.

**Figure 3:**
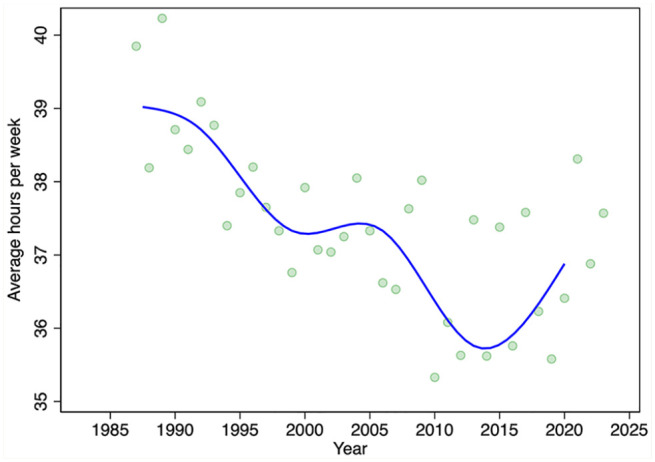
Average weekly hours worked by pharmacists, by year, 1987–2023 Green markers are annual estimates; the superimposed blue line is the median spline curve fit to the annual estimates. Data source: Statistics Canada Labour Force Surveys.^14^

**Figure 4:**
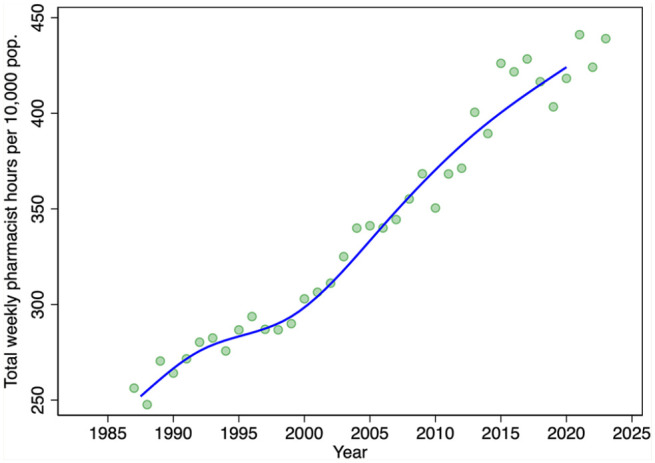
Total hours pharmacists worked each week per 10,000 population, by year, 1987–2023 Total hours pharmacists worked each week per population is calculated as the average weekly hours per pharmacist times the number of pharmacists divided by the population. Green markers are annual estimates; the superimposed blue line is the median spline curve fit to the annual estimates. Pop., population. Data source: Total pharmacists: 2001-2023: NAPRA.^9^ Pre-2001: Canadian Institute for Health Information and Health and Welfare Canada.^11-13^ Average weekly hours per pharmacist: Statistics Canada Labour Force Surveys 1987–2023.^14^ Population: Statistics Canada Table: 17-10-0005-01.^18^

We turn next to evidence on the average hours of labour supplied from the LFS. Despite the variation in annual estimates of average hours worked per week, there is a clear downward trend in the average hours of labour supplied over the period 1987 to 2015 (Figure 3). Hours trend upward after 2015. Thus, prior to 2015, there was an increasing number of pharmacists, each working fewer and fewer hours. After 2015, the number of pharmacists stabilized but average hours worked increased. On net, the total hours pharmacists collectively worked per week per 10,000 population over the period 1987 to 2023 increased (Figure 4).

**Figure 5:**
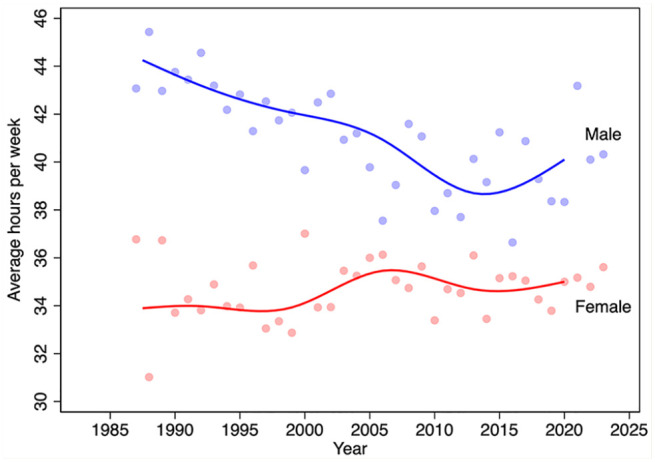
Average weekly hours worked by pharmacists, by sex and year, 1987–2023 The blue line is the median spline curve fit to the male annual estimates, shown as light blue markers. The red line is the median spline curve fit to the female annual estimates, shown as light red markers. Data source: Statistics Canada Labour Force Surveys.^14^

Figure 5 displays trends in the average hours worked per week, by sex. Males worked longer than females did each year over the period 1987 to 2023. However, male hours declined until about 2013 and then increased slightly thereafter. Female hours increased slightly over this period.

**Figure 6:**
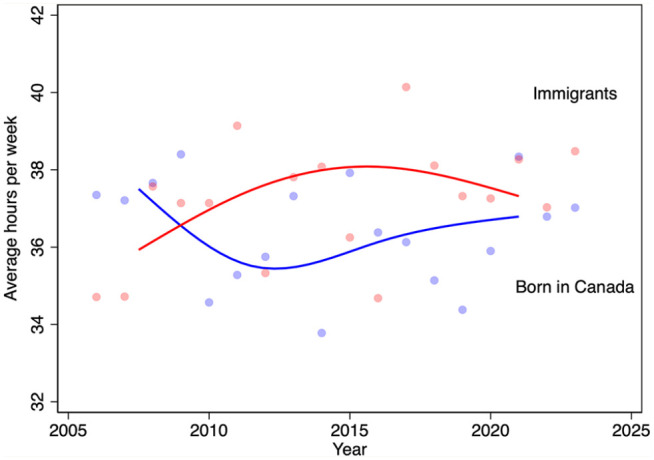
Average weekly hours worked by pharmacists, by immigration status and year, 2006–2023 The blue line is the median spline curve fit to the annual estimates for Canada-born pharmacists, shown as light blue markers. The red line is the median spline curve fit to the annual estimates for immigrant pharmacists, shown as light red markers. Data source: Statistics Canada Labour Force Surveys.^14^

We next turn to differences in trends in average weekly hours worked between pharmacists born in Canada and those born elsewhere (Figure 6). The LFS began to query immigration status in 2006, so the analysis period is not as long as for the other analyses. The data suggest diverging trends in hours worked from about 2006 to 2012, with immigrant pharmacist hours increasing and Canada-born pharmacists declining. Since 2012, however, the average hours of the 2 groups have converged.

**Figure 7:**
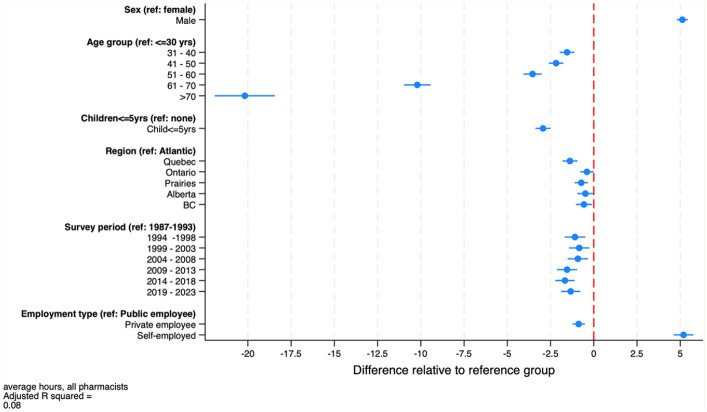
Estimates of the regression model of weekly hours worked by pharmacists, both sexes combined, 1987–2023 Blue bars are 95% confidence intervals. Data source: Statistics Canada Labour Force Surveys.^14^ BC, British Columbia.

Figure 7 displays the estimated parameters of the regression models of average hours worked per week, along with 95% confidence intervals (regression model parameter estimates also appear in Table 1). These models provide additional insights into the role of life-cycle factors (pharmacist age, sex, the presence of young children at home), among other factors, on the average weekly hours that pharmacists work. Over the survey period, we find that, controlling for age, the presence of young children at home, and other factors, males work, on average, about 5 hours longer each week than females do. Hours decline over the life cycle, dropping markedly after age 60. The presence of young children at home reduces hours by about 3 per week. Pharmacists from the Atlantic region work slightly longer hours than pharmacists elsewhere in Canada do. Mirroring the data in Figure 3, holding other factors constant, hours worked were highest in the period 1987 to 1993, then declined until 2018, after which hours increased slightly.

**Table 1:** Estimates for regression models of average weekly hours worked by pharmacists, all pharmacists combined and by sex

Variable	All	Male	Female
Male [female]	5.12181		
	0.16341		
Age group [<30 years]			
31–40	−1.54459	−1.0103	−1.67972
	0.21576	0.33902	0.27358
41–50	−2.17876	−1.16364	−3.15902
	0.21392	0.34496	0.27161
51–60	−3.53848	−1.83667	−4.86627
	0.26676	0.40206	0.35663
61–70	−10.2049	−9.43978	−10.22758
	0.39555	0.53335	0.59318
>70	−20.17619	−19.57131	−18.09202
	0.88722	1.08778	1.54272
Child ≤5 years [none]	−2.93481	0.03788	−5.17871
	0.22281	0.32569	0.29847
Region [Atlantic]			
Quebec	−1.37628	−1.31412	−1.3692
	0.22189	0.36983	0.2786
Ontario	−0.39654	0.14004	−0.70859
	0.20014	0.30935	0.25967
Prairies	−0.72197	−0.09603	−1.31152
	0.19839	0.32574	0.24799
Alberta	−0.48381	0.10676	−0.88708
	0.24084	0.40006	0.30066
BC	−0.56894	0.56958	−1.4732
	0.23483	0.37086	0.2996
Survey period [1987–1993]			
1994–1998	−1.08637	−1.66514	0.05866
	0.30309	0.39868	0.4301
1999–2003	−0.83993	−1.75873	0.51115
	0.3005	0.45726	0.39919
2004–2008	−0.91708	−3.69871	1.5948
	0.30384	0.4465	0.40654
2009–2013	−1.54327	−4.15565	0.89264
	0.29688	0.45399	0.38976
2014–2018	−1.66739	−4.14011	0.67052
	0.28398	0.41509	0.38227
2019–2023	−1.33385	−3.46666	0.76297
	0.28353	0.39641	0.39446
Employment type [public employee]			
Private employee	−0.87441	−0.52132	−0.94351
	0.18361	0.3277	0.21698
Self-employed	5.19288	5.88597	4.36348
	0.29055	0.40086	0.47049
Constant	39.05126	43.68021	38.54472
	0.3137	0.49897	0.40289
Sample size	36230	15186	21044
*R*^2^ (adjusted)	0.144	0.144	0.082

There are 2 statistics presented for each covariate: The parameter estimate and the estimated standard error.

Data source: 1987–2023 Statistics Canada Labour Force Surveys.^14^

BC, British Columbia.

The regression model also includes indicators of workplace type: public, private firm employee, and self-employed. As was noted, public sector pharmacists typically work in hospitals, employees of private firms are typically staff pharmacists in community pharmacies, and the self-employed typically own a community pharmacy or operate one for a chain. The models suggest that pharmacy owners work about 5 hours a week longer and community pharmacists work about 1 hour less than hospital pharmacists do.

**Figure 8:**
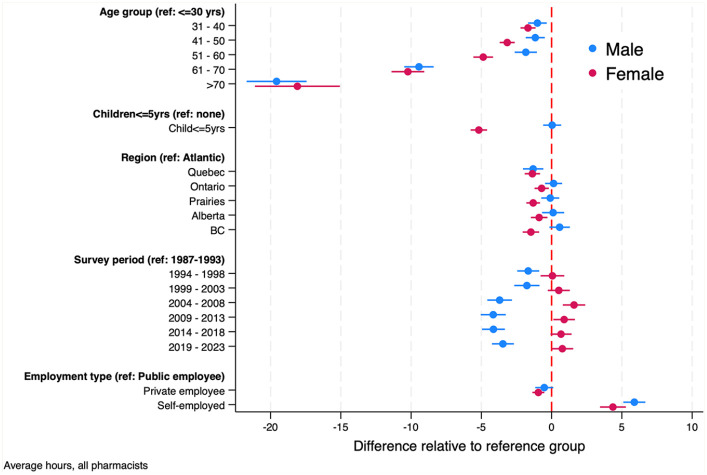
Estimates of the regression model of weekly hours worked by pharmacists, by sex, 1987–2023 The blue bars are 95% confidence intervals for the parameters of the regression models of male average weekly hours. The red bars are 95% confidence intervals for the parameters of the regression models of female average weekly hours. Data source: Statistics Canada Labour Force Surveys.^14^ BC, British Columbia.

Figure 8 presents the results of the sex-specific regression models of average pharmacist work hours.These estimates differed from the combined regression estimates in 3 ways. First, the impact on work hours of having children younger than 5 years varied by sex. Females with children younger than 5 years worked 5 fewer hours each week compared with females who did not have children under 5 years at home. The impact of young children at home had no material effects on the average hours that male pharmacists worked. Second, the time trends in hours worked by sex, displayed in Figure 5, persisted in the regression estimates. Females’ hours have increased modestly over time and males’ hours decreased, although they started from different baselines. Third, there was less regional variation in males’ hours worked than there was for females. The only regional difference in hours worked by male pharmacists was between Quebec and the rest of Canada, with Quebec pharmacists working about 2 fewer hours per week.

**Figure 9:**
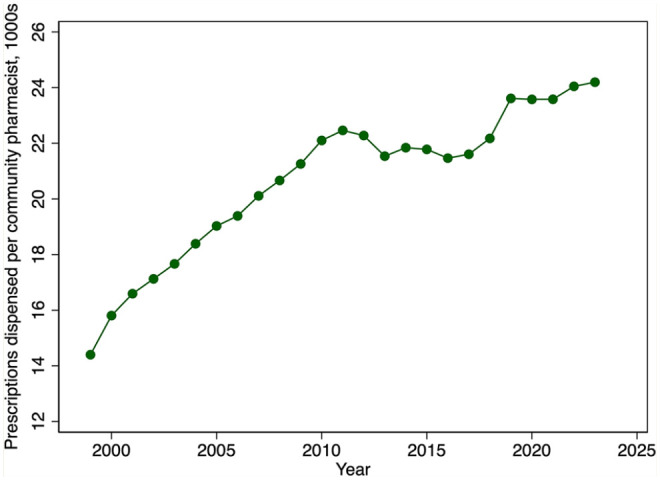
Prescriptions dispensed per community pharmacist, in 1000s, by year, 1999–2023 Data source: community pharmacists, 2001–2023: NAPRA.^9^ Prescription volume: IQVIA Compuscript surveys 1999–2023.^16^ 1999–2000: Community Pharmacy Trends Report by Healthcare and Financial Publishing, a division of Rogers Media.^17^

**Figure 10:**
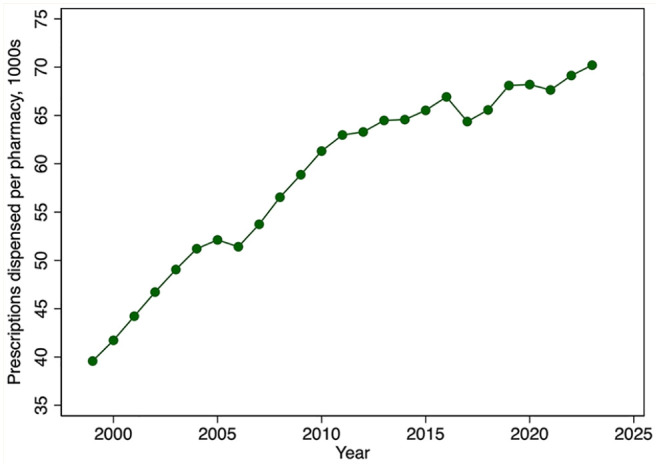
Prescriptions dispensed per community pharmacy, in 1000s, by year, 1999–2023 Data source: Community pharmacies: 2001–2023: NAPRA.^9^ Prescription volume: IQVIA Compuscript surveys 1999–2023.^16^ 1999–2000: Community Pharmacy Trends Report by Healthcare and Financial Publishing, a division of Rogers Media.^17^

Figure 9 provides evidence on trends in community pharmacist productivity. The figure reveals a marked growth in the number of prescriptions dispensed per community pharmacist from 1999 until 2011. This growth is especially remarkable given the decline in the average weekly hours that pharmacists worked over this period. Community pharmacist productivity then plateaued until about 2019, after which it again increased modestly. There have also been steady increases in the number of prescriptions dispensed per community pharmacy from 1999 until 2011, after which the rate of growth declined (Figure 10).

## Discussion

In this article, we provide evidence on various aspects of the supply of pharmacist labour in Canada over the past 4 decades. We find sustained growth in the number of pharmacists relative to the population. The Royal Commission on Health Services reported 4.98 pharmacists per 10,000 population in 1960.^4^ Pharmacy density increased slowly over the next 2 decades—there were 5.9 pharmacists per 10,000 population in 1982—but density then doubled to 11.8 pharmacists per 10,000 population by 2016. This growth appears primarily due to an increase in the number of graduates of pharmacy training programs in Canada and growth in the number of IPGs.

There was more modest growth in the number of community pharmacies per capita over this period, suggesting that pharmacists increasingly found employment in hospital pharmacies, the pharmaceutical industry, and other workplaces.

The growth in the number of pharmacists was offset by reductions in the average hours that pharmacists work per week over the period 1987 to 2015. This appears to be driven by 2 factors. First, the female share of the pharmacist workforce is increasing, and females tend to work fewer hours on average than do males. The second factor reducing hours worked is a reduction in the number of hours that male pharmacists work, a trend also observed among male physicians.^6,21^ Our models also found that hours worked declined with pharmacist age, with steep declines observed after age 60 years. On net, however, the total number of weekly hours worked—the average weekly hours worked per pharmacist times the number of pharmacists—has increased in both absolute and per capita terms.

IPGs comprise a large share of the pharmacist workforce. The IPG share of the national pharmacist workforce was 36% in 2023.^3^ In Ontario, the share was 49%.^3^ This suggests that the labour supply decisions of IPGs are particularly important for workforce planning. We find that pharmacists who immigrated to Canada (our proxy measure of IPG status) work about the same number of hours as pharmacists who were born in Canada do.

This expansion in the pharmacist labour supply was reinforced by an increase in pharmacist productivity, at least in the community pharmacy sector. Productivity, measured using the average number of prescriptions filled per community pharmacist, has increased markedly since 1999. Hospital pharmacist productivity likely has increased even more given the intensive hospital employment of pharmacy technicians. For example, Ontario hospitals employ 1.14 technicians for each pharmacist.

This study had some limitations. The data and analysis presented here provide basic information on the capacity of Canada’s pharmacist workforce and how this capacity is trending, but more detailed information is required for effective workforce planning. First, pharmacy and pharmacist density vary markedly by province.^3,22^ Thus, planners would need to assemble province-level workforce data. Second, our regression models of pharmacist hours revealed sharp declines in hours after age 60 years. These life-cycle effects on hours would need to be combined with data on the projected age distribution of the pharmacy workforce to accurately predict future labour supply. Third, provincial governments have granted pharmacists the authority to provide a growing number of primary care services. Pharmacist workforce planning requires forecasts of patient demand for these labour-intensive clinical services and an assessment of the capacity of pharmacists to provide them. There is evidence that community pharmacist workloads are very high and, indeed, are adversely affecting their mental health.^23,24^ Thus, some expansion in supply or other policy intervention may be warranted. Finally, the IPG share of Canada’s pharmacy workforce is approaching 40%, yet little is known about their integration into Canada’s pharmacy sector and how their skills are being used. This remains a fertile area of research.

## Conclusion

The pharmacist labour supply affects patient access to pharmacotherapy, immunization, and other primary health care services. There is little published evidence on the pharmacist labour supply in Canada, yet these data are needed to determine the capacity of government-subsidized pharmacist training programs, IPG immigration targets, and other aspects of pharmacist workforce planning. We evaluated long-term trends in the number of pharmacists in Canada, their average hours worked, and how demographic and other factors affect hours worked. We also examined trends in community pharmacist productivity. We found a marked increase in the total number of weekly hours that pharmacists work—the average weekly hours worked per pharmacist times the number of pharmacists—relative to population. Community pharmacist productivity has also increased. These data inform secular trends in the capacity of the pharmacist workforce. More detailed regional-level information, however, is required for pharmacist workforce planning. ■

## Supplemental Material

sj-pdf-1-cph-10.1177_17151635251330871 – Supplemental material for Long-term trends in the labour supply and productivity of pharmacists in Canada Supplemental material, sj-pdf-1-cph-10.1177_17151635251330871 for Long-term trends in the labour supply and productivity of pharmacists in Canada by Paul Grootendorst, Boris Kralj and Arthur Sweetman in Canadian Pharmacists Journal / Revue des Pharmaciens du Canada
