# Supplementary material for: Long-term trends in the labour supply and productivity of pharmacists in Canada
Source: Can Pharm J (Ott). 2025 Jun 11;158(4):236–46. doi: 10.1177/17151635251330871 (PMC12162522; doi:10.1177/17151635251330871)
Supplement: sj-pdf-1-cph-10.1177_17151635251330871 – Supplemental material for Long-term trends in the labour supply and productivity of pharmacists in Canada [file sj-pdf-1-cph-10.1177_17151635251330871.pdf]

APPENDIX 1 – Details on the Statistics Canada Labour Force Survey

The Statistics Canada Labour Force Survey (LFS) is a monthly household survey of a sample of individuals who are representative of the civilian, non-institutionalized, off-reserve (in the provinces) population 15 years of age or older. It is conducted nationwide, in both the provinces and the territories. The sample size of the LFS is approximately 55,000 households; data are collected on about 100,000 individuals. In addition to being the main source of timely labour market data for researchers, governments use LFS data to measure unemployment rates and to design education and training programs, retirement pensions and income support, and to determine benefit eligibility and entitlement for the Employment Insurance system.

Data collection for the LFS is carried out each month over the 10 days following the LFS reference week. Although in practice there is modest attrition, in principle the LFS methodology calls for each sampled household to remain in the sample for 6 consecutive months before being rotated out and replaced by a household in the same area or a similar one. The reference week is normally the week containing the 15th day of the month and stretches from Sunday to Saturday. LFS interviews can be conducted by telephone interviewers working out of Regional Office Computer Assisted Telephone Interview (CATI) sites or by personal visits from a Computer Assisted Personal Interview (CAPI) field interviewer. The interviewer first obtains sociodemographic information for each household member and then obtains labour force information for all members aged 15 and over. Information about all household members is usually obtained from one knowledgeable household member. Such “proxy” reporting accounts for approximately 65% of the information collected. Most subsequent monthly interviews (normally 5) are conducted by telephone. Starting in 2015, households that meet specific criteria may be offered the option of completing the subsequent surveys online.

Under Canada’s Statistics Act (https://laws-lois.justice.gc.ca/eng/acts/S-19/FullText.html), Canadians are obligated to participate in the LFS. Statistics Canada addresses this issue in its general FAQs for various survey participants at: https://www.statcan.gc.ca/en/survey/faq (under “Do I have to participate?”), and for the LFS at: https://www.statcan.gc.ca/en/survey/household/3701. Having said this, it seems likely that Statistics Canada informs Canadians of their obligation but does not always strictly enforce it.

Industry and occupation codes are assigned using the respondent’s job description on the questionnaire. The coding is performed manually and is based on the classifications described in the North American Industry Classification System (NAICS) and the National Occupational Classification (NOC) system. When a respondent holds more than 1 job or business, the job or business involving the greatest number of usual hours worked is considered to be the main job. The LFS distinguishes between usual hours worked and actual hours worked. We focus on actual hours worked. This is the number of hours actually worked by the subject during the reference week, including paid and unpaid hours. These hours reflect temporary decreases or

increases in work hours (for example, hours lost due to illness and vacation, or more hours worked due to overtime).

Approximately every 5 years, population estimates are rebased or reweighted to the most recent census population counts. The sample data are weighted to enable tabulations of estimates at national, provincial, and subprovincial levels of aggregation. The last rebasing was in 2020. To keep the sampling frame up-to-date and ensure reliable estimates, every 10 years, after the decennial population census, the LFS undergoes a sample redesign to reflect changes in population characteristics and new definitions of geographic boundaries. The last sample redesign was in 2015. Redesign of the questionnaire, data collection, processing, and dissemination systems occur approximately every 20 years. The last redesign for the questionnaire was in 1997 when several questions were added (e.g., union membership).

Historically, the response rate to the LFS has been quite good relative to comparators. For example, in 2019 it averaged 87.0%. However, during the COVID-19 pandemic (when face-to-face interviewing was paused), response rates fell. For example, the response rate averaged 69.8% in the second half of 2020 and 69.5% in 2021. It increased after face-to-face interviewing was reintroduced in November 2022.

Study sample

For our study, we identified and extracted pharmacists in the LFS using the National Occupational Classification (NOC); the NOC code for pharmacists is 3131. We included only those respondents whose main job was in an industry compatible with a practicing pharmacist. Observations with the following North American Industry Classification System (NAICS) codes were included in our sample: 6211, offices of physicians; 6214, outpatient care centres; 6220, hospitals; 6230, nursing and residential care facilities; 9120, provincial and territorial public administration; 5417, scientific research and development services; 3254, pharmaceutical and medicine manufacturing; 4451, grocery stores; 4461, health and personal care stores; 4521, department stores; and 4529, other general merchandise stores. We excluded the following observations on individuals who reported being pharmacists: 1) those who report working in an industry that does not appear relevant to their field; 2) those who report being full-time students; and 3) those below the age of 23 years of age and those above age 80. The resulting overall sample size for our study, over the 1987 to 2023 period, consisted of 34,163 pharmacists.

References

1. Statistics Canada Labour Statistics Division. Labour Force Survey, January 2020 [Canada]: Study Documentation. Ottawa, Canada. Available: https://www23.statcan.gc.ca/imdb/p2SV.pl?Function=getSurvey&Id=1316133.

2. Statistics Canada. Methodology of the Canadian Labour Force Survey. Ottawa, Canada, 2017.
3. Usalcas J, Kinack M. History of the Canadian Labour Force Survey, 1945 to 2016. Statistics Canada Catalogue no. 75-005-M – No. 2016001, 2019.
4. Statistics Canada. Guide to the Labour Force Survey 2020. Statistics Canada Catalogue no. 71-543-G, 2020.

Grootendorst P, Kralj B, Sweetman A. Long-term trends in the labour supply and productivity of pharmacists in Canada. *Can Pharm J (Ott)* 2025;158. DOI 10.1177/17151635231330871.
